# Heart Transplantation and Left Ventricular Assist Devices: Long-Term Prognosis and Effects on Mental Health

**DOI:** 10.7759/cureus.68691

**Published:** 2024-09-05

**Authors:** Hairya Ajaykumar Lakhani, Lenin Steve Lizarzaburo Penafiel, Marc Fakhoury, Melinda Seide, Patricio Xavier Duran S, Jia Whei See, Damandeep Kaur Dhillon, Shivendra Shah, Aysha Mohsin Khan, Marthand Nimmagadda, Thirumalasetty Susmitha, Manju Rai

**Affiliations:** 1 Internal Medicine, Smt. B. K. Shah Medical Institute and Research Center, Vadodara, IND; 2 Internal Medicine, Universidad de Las Americas, Quito, ECU; 3 Cardiology, Saint Joseph University of Beirut, Beirut, LBN; 4 Internal Medicine, St. George’s University School of Medicine, St. George’s, GRD; 5 Internal Medicine, Universidad de Cuenca, Cuenca, ECU; 6 Internal Medicine, Universitas Sriwijaya, Kota Palembang, IDN; 7 Internal Medicine, St. James School of Medicine, St. Vincent, VCT; 8 Internal Medicine, Nepalgunj Medical College, Nepalgunj, NPL; 9 Internal Medicine, Karuna Medical College, Palakkad, IND; 10 Internal Medicine, American University of Barbados, Bridgetown, BRB; 11 Internal Medicine, Odessa National Medical University, Odessa, UKR; 12 Biotechnology, Shri Venkateshwara University, Gajraula, IND

**Keywords:** end-stage heart failure, heart transplantation, left ventricular assist devices, long-term outcomes, mental health, patient selection

## Abstract

Heart transplantation and left ventricular assist devices (LVADs) have emerged as crucial interventions for end-stage heart failure, dramatically improving patient outcomes. This narrative review examines their historical context, indications, procedures, and outcomes, as well as their impact on long-term survival, quality of life, functional status, and mental health. While heart transplantation remains the optimal treatment, donor scarcity limits its application. LVADs have become a viable alternative, either as a bridge to transplantation or as destination therapy. Both interventions demonstrate similar long-term survival rates and significant improvements in health-related quality of life and functional status. However, they present distinct long-term management challenges, including immunosuppression needs for transplant recipients and device-related issues for LVAD patients. Mental health effects are considerable, necessitating psychological support and adaptive coping strategies. Complications such as infection, bleeding, and thrombosis remain concerns for both interventions. Patient selection criteria, technological advancements, and long-term management strategies are critical factors in optimizing outcomes. Future research should focus on device miniaturization, enhanced biocompatibility, and less invasive insertion techniques to further advance these therapies and improve patient care in end-stage heart failure.

## Introduction and background

**Figure 1:**
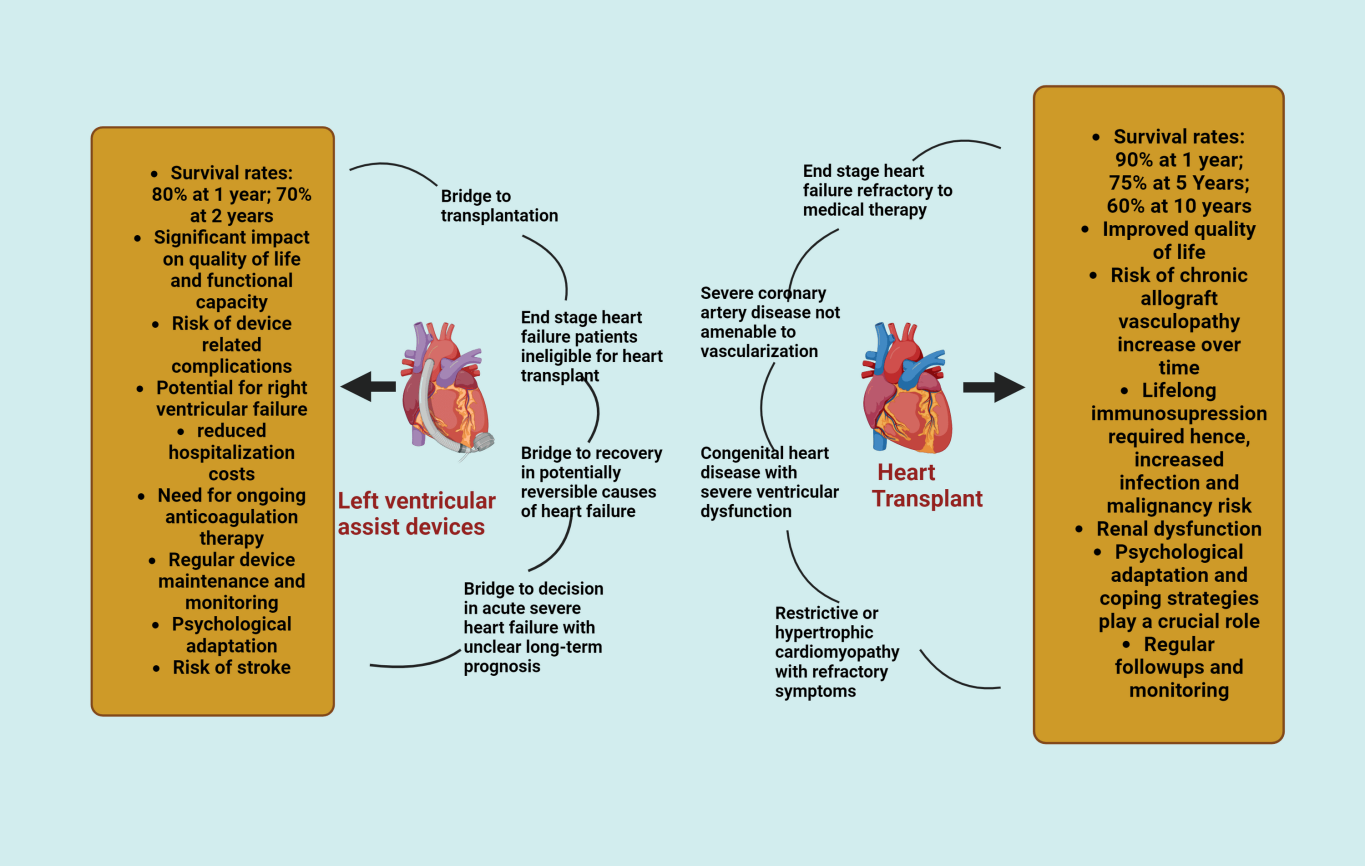
Characteristic features of heart transplantation and left ventricular assist devices Image credits: Aysha Mohsin Khan; created in BioRender.com

Heart transplantation and left ventricular assist devices (LVADs) are the two critical interventions effectively carried out for end-stage heart failure for the past three decades (Figure 1) [1]. This has significantly improved survival rates and quality of life of patients presenting with end-stage symptoms. Although it has significantly improved the functional capacity of the recipients, the post-operative complications and certain psychological challenges faced by patients are also an inevitable part of the process.

**Figure 2:**
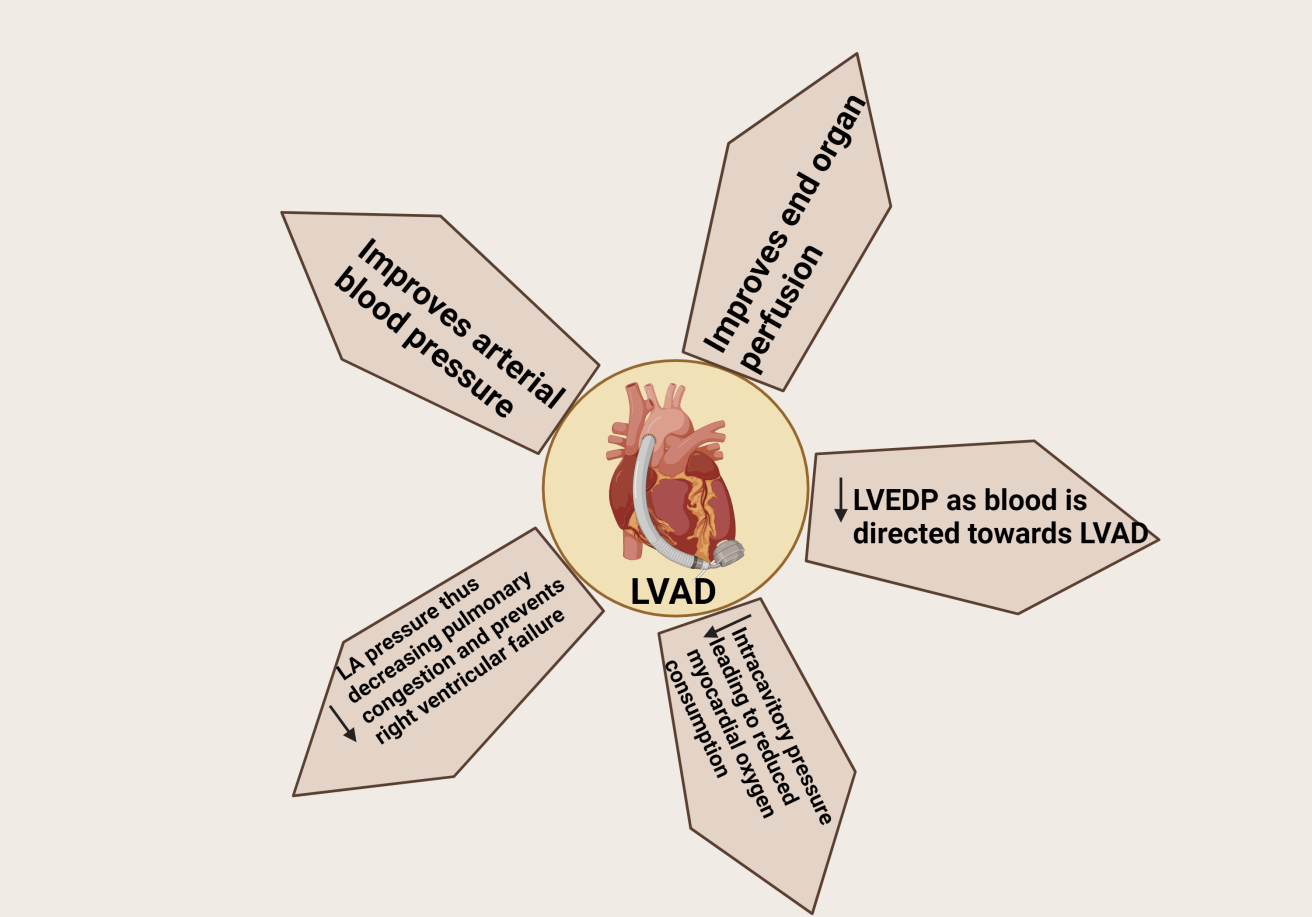
Mechanism of LVAD support for the heart LVAD: Left ventricular assist device; LVEDP: left ventricular end-diastolic pressure; LA: left atrial. Image credits: Marthand Nimmagadda; Created in BioRender.com

Since the inception of heart transplantation, over 120,000 individuals have received this life-saving treatment. However, the scarcity of donor organs remains a significant challenge for both patients and healthcare providers [2]. As a result, mechanical circulatory support devices such as LVADs have emerged as an excellent alternative for patients too ill to await a donor heart [3]. These devices assist ventricular blood flow and serve as a bridge to transplantation, destination therapy, or recovery (Figure 2) [1].

The history of mechanical circulatory support dates back to 1960 when Dr. William Pierce of Penn State University implanted the first corporal VAD [4]. Pierce later developed a pneumatic cardiac support device that gained FDA approval as the first bridge-to-transplant device and has been used in over 4,000 patients [5]. A significant milestone was achieved in 1966 when Liotta and De Bakey performed the first successful LVAD implantation, providing circulatory assistance to a patient in cardiogenic shock following cardiac surgery [5]. The following year, in 1967, Christian Bernard conducted the first successful human heart transplantation in Cape Town, South Africa, marking a new era in cardiac care [2].

Early LVAD designs intended to promote cardiac function using a physiological 1/3 systole/2/3 diastole cycle [2]. However, these pulsatile pumps had size and weight constraints, which led to the creation of continuous flow designs. Current technologies provide various benefits based on the patient's demands and are constantly updated to improve patient outcomes, reduce problems, and increase durability. Some popular LVADs include Heart Mate 2, Heart Mate 3, Heart Ware, and Jarvik 2000.

This narrative review aims to explore the evolution of heart transplantation and the introduction of LVADs in the treatment of end-stage heart failure. The review will provide a comprehensive overview of heart transplantation and LVAD procedures, including their indications, post-operative care protocols, and potential complications. Furthermore, it will evaluate the impact of these interventions on both short-term and long-term patient survival rates, as well as assess their effects on patients' quality of life. By addressing these objectives, this review seeks to offer a thorough understanding of current practices in advanced heart failure management, highlighting the benefits and challenges associated with heart transplantation and LVAD therapy.

## Review

Methods

We performed a comprehensive literature search using electronic databases including PubMed, MEDLINE, and Google Scholar. The search terms included combinations of keywords such as "heart transplantation," "left ventricular assist devices," "LVAD," "end-stage heart failure," "long-term outcomes," "quality of life," "mental health," and "psychological effects." Articles published in English between 2000 and 2024 were considered for inclusion. We focused on peer-reviewed original research articles, systematic reviews, meta-analyses, and clinical guidelines. Case reports and small case series were generally excluded unless they provided unique insights.

Relevant information was extracted from the selected articles, including data on patient selection criteria, surgical techniques, post-operative care, long-term survival rates, quality of life outcomes, and psychological effects. We synthesized this information to provide a comprehensive overview of the current state of heart transplantation and LVAD therapy, highlighting both the benefits and challenges associated with these interventions. The quality of the included studies was assessed based on their methodological rigor, sample size, and relevance to the review's objectives. However, given the narrative nature of this review, a formal quality assessment tool was not applied.

Heart transplantation

A heart transplant (HT) is one of the treatments for end-stage heart failure. It is one of the treatments to prolong the life of the patients if they are suffering from stage D heart failure (most advanced stage of heart failure). It is indicated when all the other medical treatments are optimized, and the surgical options provide no benefit [6]. The study by Guglin et al. discussed the parameters that determine the eligibility of a patient for an HT [7]. The study indicated that the initial phase of evaluation involves determining whether the patient's response to the accepted medical treatment for an HT or LVAD should be taken into account. Other factors for patient selection criteria are left ventricular ejection fraction below 25%; multiple instances of hospitalization due to heart failure within the last six months; non-responsiveness to cardiac resynchronization therapy; and inability to follow prescribed medical treatment due to a lack of tolerance for guidelines.

An HT is typically limited to younger patients and those with a body mass index (BMI) below 35 kg/m^2^. It is contraindicated for patients with pulmonary hypertension as the right ventricle of the heart may not be able to cope with the increased pressure in the lungs and could potentially fail. Malignancy or any ongoing infections are also considered contraindications for HTs due to the increased risk of developing a malignant growth caused by post-transplant immunosuppression [7].

Psychosocial and behavioral aspects should be taken into account when choosing patients, in addition to clinical or radiological risk factors. A study by Jünger et al. (2005) found that individuals with depression had a greater chance of transplantation or a combined endpoint of transplantation and mortality than patients without depression [8]. It is critical to evaluate the patient's support network, coping skills, and history of substance misuse and mental illness [6].

Quick suicide attempt depression is associated with an increased risk of post-transplant death and morbidity [9]. As a result, it is critical to provide immediate psychological support for effectively managing stress and anxiety after the transplant. To avoid rejection of the transplanted organ, it is critical to adhere to the specified immunosuppressive medication regimen and attend frequent clinic sessions [6]. Corticosteroids and calcineurin inhibitors, such as tacrolimus and cyclosporine, are often prescribed for post-operative care. Because transplant rejection and infection are more likely, the post-transplant phase is very crucial. Effective risk mitigation can be achieved by vigilant patient monitoring. Enhancing the overall quality of life in post-surgery conditions requires efficient management of stress and anxiety [10]. One of the main challenges following an HT is antibody-mediated or cellular rejection, thus continuous monitoring is necessary for minimizing these risks [11]. Steroids, cortex, and diesel depletion therapy are the principal treatments for cellular rejection. To address these hazards, routine monthly endocardiograms, gene expression, profiling, and self-free DNA testing are performed. Among HT patients, cardiac allograft vasculopathy is one of the leading causes of death during the first year of the transplant. To lower the incidence of mildly heard renal impairment following HT, adjustments and immunosuppressive regimens should be made. For maintenance following transplantation, a lifetime of immunosuppressive therapy is required [11].

Left ventricular assist devices

Indication and Patient Selection

**Figure 3:**
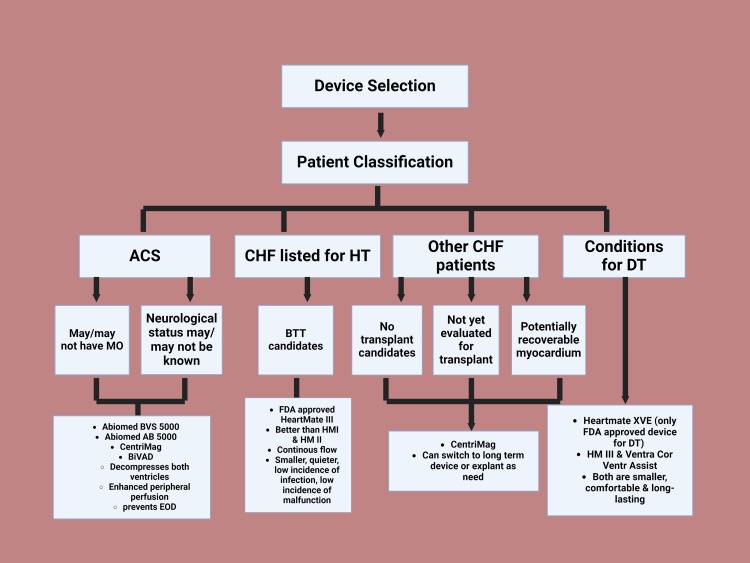
Device selection ACS: Acute cardiogenic shock; CHF: chronic heart failure; HT: heart transplant; DT: destination therapy; MO: multiorgan failure; BTT: bridge to transplant Image credits: Thirumalasetty Susmitha, created in BioRender.com

In recent times, LVADs have gained popularity as a bridge to HT or even as a stand-alone treatment for those not eligible for an HT [12]. The majority of patients cannot receive HT due to the increasing incidence of advanced heart failure (AHF) patients and the scarcity of donor hearts [13]. Since HT is not an option, contemporary LVADs are therefore preferred as medical treatment in advanced stages due to their much higher overall survival and quality of life [14]. Evaluation is required prior to contemplating DT-LVAD candidacy (Figure 3).

When evaluating patients for LVAD implantation, several crucial factors must be considered. Unlike heart transplantation, LVAD implants have no age restriction, making them viable for elderly individuals. Older patients with heart failure and reduced ejection fraction (HFrEF) often show quicker recovery in physical and functional status post-LVAD implantation [15]. Obesity and diabetes, major cardiovascular comorbidities in AHF, affect about one-third of patients [12]. While diabetes may be viewed as a relative contraindication due to poor long-term outlook, obesity is no longer considered a contraindication [16]. Weight management strategies, including cardiac rehabilitation and psychosocial support, may be considered [17]. Chronic kidney disease (CKD) is common in AHF patients, with over 30% experiencing microalbuminuria or macroalbuminuria. Severe renal insufficiency may benefit from specific therapeutic approaches [16].

Hepatic dysfunction, similar to renal dysfunction, is observed in heart failure patients due to inadequate organ perfusion and venous congestion. However, the liver shows a superior capacity for reversibility. While liver fibrosis isn't a formal contraindication, further biopsy stratification information is needed. Bilirubin is the recommended test for liver function assessment [18], with "irreversible" liver impairment considered a legal contraindication [16]. Pre-LVAD implantation, optimizing lung function through respiratory physiotherapy and aggressive treatment of pulmonary edema, is recommended [17]. Patients with renal cell and hematological malignancies have the worst prognosis. Neurological and cognitive functions should be carefully evaluated before DT-LVAD implantation, with transient ischemic attacks or cerebrovascular accidents being exclusion criteria [19]. Active substance abuse or systemic infections, including endocarditis, are contraindicated for LVAD [16,18,20]. Table 1 provides a comprehensive overview of indications and patient selection criteria for LVADs.

**Table 1:** Indications and patient selection criteria for left ventricular assist devices (LVADs) HbA1c: Glycated hemoglobin; BMI: body mass index; CKD: chronic kidney disease; eGFR: estimated glomerular filtration rate; PVD: peripheral vascular disease

	Inclusion criteria	Relative contraindications	Absolute contraindications
Age [15]	No age limit. consider carefully for 70+, focus on physical/functional status.		
Cardiovascular risk factors [12]	Controlled diabetes (HbA1c < 7%), BMI ≤ 35, managed valvular diseases/shunts, treatable intracardiac thrombus.	HbA1c 7.5-8%, BMI 35-40, end-organ complications.	HbA1c > 8%, BMI > 40, untreatable valvular diseases/thrombus.
Renal function [16]	eGFR > 30 mL/min/1.73 m², stable function.	eGFR < 30 mL/min/1.73 m² (not on dialysis), rapid decline.	eGFR < 30 mL/min/1.73 m² on dialysis, irreversible damage.
Liver function [18]	Bilirubin ≤ 2 mg/dL	Bilirubin 2-3 mg/dL	Bilirubin > 3 mg/dL
Medical background [17,19]	Stable malignancies (>1 year survival), managed comorbidities.	PVD, mild coagulopathies, malignancies requiring monitoring.	Active infection, substance abuse, malignancies (<1 year survival).
Fragility and basal situation [20]	Manageable, potential for improvement, adequate cognitive function.	Moderate, requiring optimization, mild cognitive impairment.	Severe without improvement potential, significant cognitive impairment.

Types and Advancements in LVAD Technology

LVADs have seen significant technological advancements over time. The evolution began with first-generation pulsatile pumps like Novacor® and HeartMate VE®, progressing to the continuous flow mechanisms of second and third-generation LVADs. Second-generation pumps, including the axial pump design of HeartMate II®, Berlin Heart INCOR®, and Jarvik 2000®, offered notable improvements in size and noise reduction compared to their predecessors. Currently, third-generation LVADs are the most widely utilized. The HeartWare® (HVAD), featuring a hybrid centrifugal flow, demonstrated comparable performance to HeartMate II® in the ENDURANCE trial and proved to be feasible for exchange in practice [21,22]. However, it was associated with significantly higher stroke rates, both ischemic and hemorrhagic, compared to HeartMate II® [18]. This highlighted the crucial need for intensive blood pressure control post-implantation, as it was identified as an independent and strong risk factor [16]. Table 2 provides a comprehensive overview of the technology employed, along with the advantages and disadvantages of LVADs across all three generations.

**Table 2:** Characteristics of first, second, and third generation of LVADs LVADs: Left ventricular assist devices

LVADs	Technology	Advantages	Disadvantages
First generation [18]	Pulsatile flow	Mimics natural heart function and immediate support.	Large size, high infection rate, mechanical failures, and limited durability.
Second generation [21]	Continuous flow with axial pumps	Smaller, lower infection rates, improved survival, better symptom management - Better management of severe heart failure symptoms	GI bleeding, thrombosis, and infection risk from external power.
Third generation [22]	Continuous-flow technology with centrifugal flow pumps	Longer durability, easier placement, lower stroke risk, and better quality of life.	Complex, expensive, requiring specialized training, and external power dependence.

Long-Term and Short-Term Outcomes of LVADs

LVAD implantation is associated with significant post-operative risks. A study by Shah et al. revealed a fivefold increase in the risk of readmission due to gastrointestinal bleeding within 60 days following LVAD implantation [23]. Older patients and those with a history of bleeding during the implantation procedure are particularly vulnerable, indicating a need for close monitoring and careful anticoagulation management to prevent rebleeding [24]. Another major challenge in long-term mechanical circulatory support is pump thrombosis in durable continuous-flow pumps [25]. Earlier data from the Interagency Registry for Mechanically Assisted Circulatory Support (INTERMACS) identified an increasing risk of pump thrombosis in recent years with the HeartMate II (HMII) left ventricular assist device.

Gosev et al. conducted a comparative study of sternal sparing (SS) and traditional sternotomy (TS) surgical approaches for HeartMate 3 (HM3) LVAD implantation [26]. The study, involving 105 patients, showed that 39% of implants used the SS method, while 61% employed the TS technique. The SS cohort, consisting of younger patients, demonstrated significantly better outcomes, including reduced rates of severe right ventricular failure, fewer blood product transfusions, and shorter hospital stays. Moreover, the SS group achieved an impressive six-month survival rate of 93%. While these findings suggest that the SS approach may be a safe and effective surgical technique for HM3 LVAD implantation in carefully selected patients, the authors emphasize the need for further research to confirm these conclusions.

Long-term prognosis

Comparison of Long-Term Survival Rates Between Heart Transplantation and LVADs

Studies have shown that the long-term survival rates between heart transplantation and LVADs have no particular difference in patients that are conducted on 1-, 2- and 5-year mortality rates [27,28]. However, there is one research that concludes differently, i.e., hospital mortality is higher for heart transplantation patients on the waiting list in comparison with BTT (bridge to transplant) LVAD patients [29].

Eighty percent of patients who underwent LVAD implantation were alive one year after the procedure, and 70% made it to the second year [30,31]. A comparable survival trend was reported by the European registry for patients with mechanical circulatory support, although to a slightly lesser extent [32]. It should also be noted that the introduction of a turbodynamic-LVAD played a significant role in improving the mortality rate as compared to the older volume displacement-LVAD that displayed inferior outcomes [33]. The newer device works under a steady rotational speed and thus less pulsatility [34], whereas the old one was designed to mimic the native ventricle [35]. Cowger et al. found that low-post-operative risk patients had an 83% one-year survival, whereas high-risk patients showed only 58% [36]. 

On the other hand, traditional HT yielded excellent results with 10.7 years of median survival and 13.6 years for first-year survivors [37]. Five-year survival was observed to be around 60% [38]. It is also important to highlight that patients scheduled for an HT who were bridged with LVADs showed a surprising 60% decrease in mortality after one year, reflecting the protective capacity of this technique [35].

Complications and Challenges in Long-Term Management

Standing in view of long-term management, patients with heart transplantation are required to maintain a regimen of immunosuppressive therapy to reduce the rate of rejection and dependency on medication in order to relieve comorbidities. A combination of a healthier lifestyle regimen, consistent pharmacological treatment, and high-quality medical care can significantly improve survival rates and enhance the quality of life for HT recipients [39]. Healthcare programs can be led by well-trained interdisciplinary teams on management post-HT. There is also the application of internet-based (eHealth) systems that is well encouraged to apply as its outcome leans positively on the improvement of lifestyle and pharmacology treatment [39]. 

Whereas for LVAD patients, the LVAD speed requires adjustment to suit the patient’s hemodynamics [40]. Mechanisms of the device must be well understood to prevent complications. Patients should also take a physical exam to verify the presence of LVADs and the arterial pulse by Doppler and if absent, the ACLS protocol should also be done with extra caution [40]. Pharmacological treatment with LVADs such as ACE inhibitors, digoxin, and beta-blockers helps to optimize heart function and reverse modeling which is achievable by mechanical unloading, normalizing the neurohormonal axis, and heightening the cross-linking of collagen [41,42]. The maintenance of a healthy lifestyle is encouraged to boost the recovery of LVAD patients, which is achievable by incorporating physical and occupational therapy [43,44]. Cardiac rehabilitation also plays a role in recovery [43]. Caregivers should also be educated on post-LVAD rehabilitation [42-44].

Quality of life and functional status

The LVAD has been indicated for use in advanced heart failure patients, in an effort to ameliorate their health-related quality of life (HRQOL), functional status, and survival of patients. Noly et al. performed a retrospective cohort study on patients who received a continuous LVAD flow implant [45]. The researchers determined that the days alive out of the hospital (DAOH) post LVAD implantation is dependent on various factors, such as the patient demographics, the quantity and type of adverse effects the patient experienced, the clinical characteristics of the patient, and HRQOL. The study found that patients who were in the lower terciles of DAOH-AF (days alive out of hospital post-LVAD implantation) compared with the intermediate and higher terciles had longer lengths of stay in the hospital, lower probability of being discharged home, and remained in a rehabilitation facility, skilled nursing facility or hospice for an extended period of time. 

Maclver et al. assessed various clinical trials and found that post-LVAD, there is improvement in the quality of life from as early as 1 to three months post-surgery [46]. With regard to physical functioning, this study focused on cardiopulmonary functioning demonstrated that by three months, patients reached their peak oxygen uptake (vO2). Additionally, patients who had LVAD were able to walk the equivalent of a NYHA (New York Heart Association) functional class 1 or II patient, which is about 393 meters. Furthermore, it has been found that exercise-based cardiac rehabilitation (EBCR) post LVAD implantation has positive implications on patient recovery. The initiation of EBCR treatment in patients ranged from immediately after LVAD treatment to up to 10 months post-treatment [47]. The study reviewed six trials that consisted of 183 patients, 83% of which were males, and found that EBCR improved peak oxygen uptake (vO2). Though this study rendered great insight, the studies had small sample sizes, and they were not heterogeneous. 

Scaglione et al. performed a study to compare patients who received EBCR post-LVAD versus heart transplantation patients, and they found that it benefits both groups similarly [48]. In addition, the LVAD patients did not show signs of deterioration up to 12 months post-EBCR. This study provided some insight but many patients dropped out of the latter part of the study, which was to test the long-term durability of EBCR, which impacted the results. Patient-reported outcomes of LVAD implantation reveal a mixed impact on quality of life. While many patients experienced improvements in daily activities such as climbing stairs, gardening, and breathing, a significant number reported adverse effects including discomfort from the device's weight, limitations in physical activities, sleep disturbances, and decreased sexual intimacy [49]. These findings highlight the complex nature of living with an LVAD, emphasizing both its benefits in managing heart failure and the challenges it presents to patient's daily lives and overall well-being.

Mental health effects

By 2020, heart failure cases in the U.S. were estimated to reach eight million, with 20%-40% of these patients experiencing depression, a rate significantly higher than in the general population [50]. This depression leads to a lower quality of life, poor self-care, increased medical service use, and higher hospital readmission and mortality rates. Mental health effects are often evaluated using tools such as the Diagnostic and Statistical Manual of Mental Disorders (DSM) and quality of life assessments like the Short Form-36 (SF-36) to monitor psychological well-being and overall life satisfaction. While LVADs generally improve quality of life, diagnosing depression in these patients can be challenging due to overlapping symptoms with heart failure. Patients often avoid discussing emotional stress due to stigma, and healthcare providers may focus on treating physical symptoms, overlooking depressive ones. Depression often presents as a physical issues, leading patients to consult non-psychiatric specialists. Identifying depression in these patients is crucial to understanding how LVAD affects their mental health [50].

LVAD recipients often experience emotional and psychological stress due to device management, power source dependency, pain, reduced sleep, limited activities, and complex medication regimens [51]. The psychological response to LVAD implantation can be divided into four phases: pre-LVAD period, hospitalization phase, early adaptation phase, and late adaptation phase. These phases vary in duration among patients. Studies show significant improvements in anxiety, depression, and quality of life post-LVAD implantation, with changes observed from 30 days to 12 months [50]. Early identification of these symptoms is crucial, as incorporating psychological support can be lifesaving. Depressive and anxiety symptoms tend to improve within one year after heart surgery, while post-traumatic stress disorder symptoms may worsen [52]. Older patients and those with certain comorbidities are at higher risk for persistent symptoms and should be closely monitored.

Palliative care consultations can address both psychological and physical symptoms, with follow-ups in the weeks after implantation being beneficial for most patients [53]. Severe mental illness (SMI) was linked to worse heart failure outcomes in men but not in women [54]. Patients with SMI had higher death rates after heart failure procedures, indicating a need for more careful monitoring, particularly for men with SMI.

Coping strategies and support system

Several studies are focused on the results of well-directed counseling and psychological support and non-beneficial outcomes that can be avoided. Prehabilitation training including psychological support improved quality of life, lower rate of post-operative complications, lower hospitalization stay, and lower rate of rehabilitation settings transfers in LVAD patients [55]. Besides, an article review defined that the main psychological factors to approach and asses are social assistance, cognitive level, psychopathology, engagement with the implant, and abuse of alcohol and other substances [56]. 

On the other hand, the mental health outcomes in a long-term period show wellbeing results in patients with HT and left ventricular devices. A cross-sectional study found that self-efficacy, defined as patient’s confidence, achieved high scores in the total psychological wellbeing score in a period of five years after HT with psychological instruments as support [57]. In addition, qualitative research concluded that patient’s experience after the HT procedure were grateful in three main aspects which are new opportunities in life, challenges with therapy and side effects, and coping with religion and society support [58]. Thus, counseling and psychological support have the desired impact on patient’s mental health ability after an HT surgery and LVAD procedures.

The role of support systems and community services in promoting mental health for patients who have undergone heart transplantation or LVAD implantation is crucial. These support mechanisms can take various forms, each contributing significantly to the patient's overall well-being and recovery process. Family support plays a pivotal role in the patient's journey, particularly during outpatient visits following heart transplantation or LVAD implantation. A cross-sectional study highlighted that families scoring high in short-term family functioning were more likely to contribute positively to the patient's mental health and desired outcomes [59]. This underscores the importance of nurturing a supportive family environment throughout the recovery process. Peer counseling and support networks have also been identified as valuable resources in the path to recovery. A case series demonstrated that peers, colleagues, caregivers, and family members can significantly contribute to and facilitate the recovery process following heart transplantation or other cardiac procedures [60]. These support systems provide patients with relatable experiences and practical advice, helping them navigate the challenges of their new reality. Psychological support emerges as a backbone in the patient's support structure. A review concluded that timely psychotherapeutic interventions, particularly when offered in the perioperative period, could effectively reduce and prevent mental health symptoms [61]. This highlights the importance of integrating psychological care into the standard treatment protocol for HT and LVAD patients.

Current scenario and future directions

The field of cardiac surgery is experiencing a continuous evolution, with minimally invasive techniques, robotically assisted coronary bypass surgery, and hybrid coronary revascularization procedures emerging as promising approaches in managing valvulopathies and ischemic coronary disease [62]. Given the systemic effects of the sympathetic system and the frequent occurrence of sympathetic reinnervation in patients, the routine use of β-blockers may offer potential benefits. This hypothesis warrants further investigation through prospective studies or randomized trials. In 2018, the International Society for Heart and Lung Transplantation issued a consensus statement aimed at standardizing psychosocial evaluations across advanced heart failure programs. Additionally, enhanced understanding of hemodynamics and end-organ perfusion has driven research into improving the functionality of rotary LVADs [63].

The rapid advancement of LVAD technologies and innovations is significantly improving treatment outcomes and patient quality of life. Key developments include transcutaneous energy transfer systems, centrifugal pumps, biocompatible materials, anti-infection coatings, longer-lasting batteries, wearable chargers, remote monitoring capabilities, AI-driven data analysis, combined regenerative therapies, less invasive implantation techniques, third-generation continuous flow models, and hemodynamic optimization. Ongoing research into miniature and partial-support LVADs aims to promote early intervention and long-term use in heart failure treatment [63]. However, the field faces challenges such as study variability, insufficient data, treatment adherence issues, clinical implementation hurdles, and financial, logistical, ethical, and regulatory concerns. Despite these obstacles, advancements in materials, design, energy management, and monitoring technologies hold the promise of transforming advanced heart failure treatment in the future.

## Conclusions

HT and LVADs have greatly improved the treatment of end-stage heart failure in the last thirty years, leading to considerable improvements in survival rates and quality of life. Notwithstanding their achievements, these therapies are accompanied by challenges such as post-operative complications and psychological issues. The limited availability of donors continues to be a significant barrier, therefore making LVADs an essential alternative for patients who are waiting for a transplantation. Progress in LVAD technology has enhanced outcomes and broadened the criteria for older and more vulnerable patients to be eligible. Both HT and LVADs necessitate meticulous patient selection and administration to minimize risks and enhance long-term effectiveness. Continual research and innovation are crucial for improving these therapies, minimizing complications, and improving the quality of life for patients.
